# Characterization of the promoter and extended C-terminal domain of Arabidopsis WRKY33 and functional analysis of tomato WRKY33 homologues in plant stress responses

**DOI:** 10.1093/jxb/erv221

**Published:** 2015-05-11

**Authors:** Jie Zhou, Jian Wang, Zuyu Zheng, Baofang Fan, Jing-Quan Yu, Zhixiang Chen

**Affiliations:** ^1^Department of Horticulture, Zijingang Campus, Zhejiang University, Yuhangtang Road 866, Hangzhou 310058, China; ^2^Department of Botany and Plant Pathology, 915W. State Street, Purdue University, West Lafayette, IN 47907-2054, USA; ^3^ Present address: Salk Institute for Biological Studies, 10010 North Torrey Pines Road, La Jolla, CA 92036, USA

**Keywords:** Arabidopsis, C-terminal domain, plant stress, promoter, response, tomato, WRKY33.

## Abstract

Arabidopsis AtWRKY33 plays a critical role in broad plant stress responses. Whether there are evolutionarily conserved homologues of AtWRKY33 in other plants and what make AtWRKY33 such an important protein in plant stress responses are largely unknown. We compared AtWRKY33 with its close homologues to identify AtWRKY33-specific regulatory and structural elements, which were then functionally analysed through complementation. We also performed phylogenetic analysis to identify structural AtWRKY33 homologues in other plants and functionally analysed two tomato homologues through complementation and gene silencing. AtWRKY33 has an extended C-terminal domain (CTD) absent in its close homologue AtWRKY25. Both its CTD and the strong pathogen/stress-responsive expression of AtWRKY33 are necessary to complement the critical phenotypes of *atwrky33*. Structural AtWRKY33 homologues were identified in both dicot and monocot plants including two (SlWRKY33A and SlWRKY33B) in tomato. Molecular complementation and gene silencing confirmed that the two tomato WRKY genes play a critical role similar to that of AtWRKY33 in plant stress responses. Thus, WRKY33 proteins are evolutionarily conserved with a critical role in broad plant stress responses. Both its CTD and promoter are critical for the uniquely important roles of WRKY33 in plant stress responses.

## Highlight

We demonstrate that WRKY33 proteins are evolutionarily conserved and play a critical role in broad plant stress responses. Both C-terminal domain and specific promoter are involved in WRKY33-induced stress tolerance.

## Introduction

WRKY proteins are sequence-specific DNA-binding transcription factors that were first identified and have since been found almost exclusively in plants (Eulgem *et al.*, 2000; Rushton *et al.*, 2010). WRKY proteins are characterized by the presence of the highly conserved WRKY domain, which contains the hallmark heptapeptide WRKYGQK and a Cx_4-5_Cx_22-23_HxH or Cx_7_Cx_23_HxC zinc-finger motif (Rushton *et al.*, 2010). WRKY proteins are encoded by large genes families with more than 70 members in *Arabidopsis thaliana* (Arabidopsis) and can be classified into groups based on the number and structure of the conserved WRKY zinc-finger motifs (Eulgem *et al.*, 2000). The first group contains two Cx4Cx22-23HxH zinc-finger motifs whereas the second and third groups contain one Cx4-5Cx23HxH and one Cx7Cx23HxC zinc-finger motif, respectively. Group II WRKY proteins have been further divided into five subgroups (IIa, IIb, IIc, IId and IIe) based on the amino acid sequences of the WRKY domains (Zhang and Wang, 2005; Rushton *et al.*, 2010). A single Group I WRKY gene has been found in the non-photosynthetic slime mould *Dictyostelium discoideum* and unicellular protest *Giardialamblia* eukaryotes as well as in the green unicellular algae *Chlamydomonas reinhardtii*, indicating that Group I WRKY proteins originated before the emergence of photosynthetic eukaryotes and are the ancestors to the other groups of WRKY proteins (Zhang and Wang, 2005).

Since the report of the first WRKY protein two decades ago, a large number of studies have focused on functional analysis of plant WRKY transcription factors. Expression of many WRKY genes is strongly and rapidly induced by pathogens and environmental stimuli, suggesting their involvement in plant responses to both biotic and abiotic stresses (Hara *et al.*, 2000; Dong *et al.*, 2003; Turck *et al.*, 2004; Eulgem, 2006; Eulgem and Somssich, 2007). Direct functional analysis through gene disruption, silencing or overexpression has confirmed that plant WRKY transcription factors play important roles in a broad spectrum of biological processes, particularly in plant disease resistance and stress tolerance (Eulgem, 2006; Eulgem and Somssich, 2007). Intriguingly, the important roles of many WRKY genes in plant disease resistance or stress tolerance have been inferred from their overexpression in transgenic plants as their loss-of-function knockout or knockdown mutants often display little or minor phenotypes (Eulgem *et al.*, 1999; Chen and Chen, 2002; Li *et al.*, 2004, 2006; Zheng *et al.*, 2007; Kim *et al.*, 2008; Lai *et al.*, 2008; Zhang *et al.*, 2008; Peng *et al.*, 2010; Shen *et al.*, 2012). These observations suggest that many WRKY transcription factors are regulators of plant disease resistance and stress tolerance through modulating or fine-tuning plant responses to specific biotic and abiotic stresses. In addition, as WRKY proteins are encoded by a gene superfamily, there is extensive functional redundancy among WRKY genes (Journot-Catalino *et al.*, 2006; Xu *et al.*, 2006; Kim *et al.*, 2008). AtWRKY33, a member of the ancestral Group I WRKY transcription factors from Arabidopsis, is an exception in that its knockout mutants are strongly compromised not only in plant resistance to necrotrophic pathogens but also in tolerance to abiotic stresses (Zheng *et al.*, 2006; Jiang and Deyholos, 2009; Li *et al.*, 2011).

We have previously shown that disruption of *AtWRKY33* severely compromises while overexpression of the gene enhances resistance to necrotrophic fungal pathogens *Botrytis cinerea* and *Alternaria brassicicola* (Zheng *et al.*, 2006). No enhanced susceptibility to the necrotrophic pathogens is observed in the mutants for *AtWRKY25* or *AtWRKY26*, two close and pathogen-responsive homologues of *AtWRKY33*. The molecular basis for the critical role of AtWRKY33 in plant resistance to necrotrophic pathogens has also been extensively investigated. AtWRKY33 interacts with ATG18a, a critical component of autophagy and *Botrytis*-induced expression of ATG18a and formation of autophagosomes are compromised in the *atwrky33* mutant plants (Lai *et al.*, 2011b). Thus, AtWRKY33 is a positive regulator of pathogen-induced autophagy, which plays a critical role in plant resistance to necrotrophic fungal pathogens (Lai *et al.*, 2011b; Zhou *et al.*, 2013). AtWRKY33 is also critical for pathogen-induced camalexin and ethylene biosynthesis (Mao *et al.*, 2011; Birkenbihl *et al.*, 2012). The high susceptibility of the *atwrky33* mutants to necrotrophic pathogens is also associated with reduced expression jasmonic acid (JA)-regulated genes such as *PDF1.2* and increased expression of salicylic acid (SA)-regulated *PR* genes, indicating positive regulation of the JA pathway but negative regulation of the SA pathway by AtWRKY33 in *Botrytis*-infected plants (Zheng *et al.*, 2006). Global gene expression profiling has revealed that down-regulation of JA-mediated gene expression in *atwrky33* mutants is associated with activation of genes encoding jasmonate ZIM-domain repressors of the JA-response pathway (Birkenbihl *et al.*, 2012). The global gene expression profiling has further revealed other genes including those involved in redox homeostasis as potential direct target genes of AtWRKY33 (Birkenbihl *et al.*, 2012). Thus, the critical role of AtWRKY33 in plant resistance to necrotrophic pathogens is linked with its positive role in the regulation of genes involved in autophagy, camalexin and ethylene biosynthesis, JA response and redox homeostasis.

Other studies have shown that AtWRKY33 are critical for plant tolerance to abiotic stresses. Null mutants for *AtWRKY33*, but not for *AtWRKY2*5, were sensitive to NaCl based on assays of both roots growth inhibition and electrolyte leakage (Jiang and Deyholos, 2009). Assays of seed germination and small seedling survival at 48°C have shown that while *atwrky33*, *atwrky25* and *atwrky26* single mutants exhibited relatively minor phenotypes in heat tolerance, the double and triple mutants for the close homologues displayed reduced germination, decreased survival and increased electrolyte leakage under heat stress (Li *et al.*, 2011). These results indicate redundant roles of the three WRKY genes in plant heat tolerance. As will be described in this report, *atwrky33* single mutants are highly sensitive to heat stress when assayed at 45°C using both seedlings and mature plants. Therefore, AtWRKY33 plays critical roles not only in plant immune systems against necrotrophic pathogens but also in plant responses to a spectrum of abiotic stresses.

As a critical regulator of plant responses to both biotic and abiotic stresses, AtWRKY33 is subject to regulation by environmental stimuli. The transcript levels of *AtWRKY33* are usually low in healthy plants but are induced rapidly and strongly in response to defence-associated stimuli and different kinds of plant pathogens (Zheng *et al.*, 2006; Lippok *et al.*, 2007). In the promoter of *AtWRKY33*, a set of three WRKY-recognized *cis*-acting DNA sequences (W boxes) is critical for pathogen- or pathogen elicitor-induced *AtWRKY33* expression (Lippok *et al.*, 2007). Chromatin immunoprecipitation using an anti-all-WRKY antibody detected increased binding of WRKY proteins to the *AtWRKY33* promoter upon treatment with benzothiadiazole S-methylester, a potent SA analog (Lippok *et al.*, 2007). A more recent study has further revealed that AtWRKY33 is activated upon phosphorylation by stress/pathogen-responsive MITOGEN-ACTIVATED PROTEIN KINASE 3 and 6 (MPK3/6) and activated AtWRKY33 activates its own expression, thereby generating a potential positive feedback mechanism for rapid and strong induction of AtWRKY33 target genes, including camalexin biosynthetic genes such as *CYP71A3* and *PAD3* (Mao *et al.*, 2011). These results indicate that AtWRKY33 acts downstream of MPK3/MPK6 cascade in regulation of pathogen-induced defence responses.

AtWRKY33 is also regulated by interacting proteins containing a conserved FxxxVQxLTG or VQ motif (Lai *et al.*, 2011a). AtWRKY33 and closely related AtWRKY25 interact with MKS1, a VQ protein substrate of MPK4 (Petersen *et al.*, 2000, 2010; Qiu *et al.*, 2008). More recently, we have shown that AtWRKY33 also interacts with two other VQ proteins in the nucleus, SIGMA FACTOR-INTERACTING PROTEIN1 (SIB1) and SIB2 (Lai *et al.*, 2011a), which are also targeted to chloroplasts and interact with plastid-encoded plastid RNA polymerase SIGMA FACTOR1 (SIG1) (Morikawa *et al.*, 2002). MKS1, SIB1, and SIB2 bind to the C-terminal WRKY domain and stimulate the DNA-binding activity of AtWRKY33 (Lai *et al.*, 2011a). The conserved V and Q residues in the VQ motif of SIB1 are important for interaction with AtWRKY33 (Lai *et al.*, 2011a). Both SIB1 and SIB2 are induced by *Botrytis* and play an important role in plant resistance to necrotrophic pathogens. These results indicate that dual-targeted SIB1 and SIB2 function as activators of AtWRKY33 in plant defence against necrotrophic pathogens (Lai *et al.*, 2011a).

As a member of the ancestral Group I WRKY proteins with broad biological roles and dynamic and complex regulation in plant stress responses, AtWRKY33 is a useful paradigm for structural and functional evolution of plant WRKY transcription factors. AtWRKY33 shares highly similar WRKY domains with several other Group I WRKY proteins such as AtWRKY25 but the broad and strong phenotypes of compromised resistance to necrotrophic pathogens and tolerance to abiotic stresses in *atwrky33* mutants are not observed in mutants for *AtWRKY25* or other Arabidopsis WRKY genes. To address the structural basis for the uniquely important roles of AtWRKY33, we have compared AtWRKY33 with its close homologue AtWRKY25 for the ability to restore disease resistance and heat tolerance to the *atwrky33* mutants. These experiments have revealed that both the protein structure, as exemplified by the unique C-terminal domain (CTD) of AtWRKY33, and expression pattern conferred by its gene promoter are critical for the important biological roles of AtWRKY33 in plant disease resistance and stress tolerance. In addition, while the interaction between a plant and a biotrophic pathogen is often controlled by a rapidly evolving gene-for-gene mechanism (Jones and Dangl, 2006), plant resistance to necrotrophic pathogens is often polygenic (Glazebrook, 2005) and, therefore, the underlying mechanisms and involved genes might be more conserved among a broad range of plant hosts. Likewise, molecular mechanisms for plant tolerance to abiotic stresses are also polygenic in nature, involving integration of many signalling pathways for adaptive responses (Akpinar *et al.*, 2012; Chen *et al.*, 2012; Huang *et al.*, 2012; Suzuki *et al.*, 2012). As a member of the ancestral Group I WRKY proteins and a central regulator of plant stress responses, WRKY33 may function as an evolutionarily conserved coordinator in plant responses to necrotrophic pathogens and abiotic stresses. To test this, we have performed phylogenetic and sequence analysis and identified evolutionarily conserved WRKY33 homologues in tomato and rice plants. Expression analysis and gene silencing have shown that the two tomato WRKY33 homologues (SlWRKY33A and SlWRKY33B) play an important role in tomato resistance to *Botrytis*. Furthermore, both *SlWRKY33A* and *SlWRKY33B* can fully restore the disease resistance and stress tolerance to the Arabidopsis *atwrky33* mutant plants. These results indicate that WRKY33 proteins are evolutionarily conserved WRKY transcription factors with a broad and critical role in plant stress responses.

## Materials and methods

### Plant materials and growth conditions

The Arabidopsis wild-type, mutant and transgenic plants used in the study are all in the Col-0 background. The *wrky33-2* mutant has been previously described (Zheng *et al.*, 2006). Arabidopsis plants were normally grown in growth chambers at 22°C, 120 μE m^-2^ s^-1^ light with 12h light/12h darkness.

Seeds of tomato (*Solanum lycopersicum* L.) cultivar Ailsa Craig were germinated in a growth medium filled with a mixture of peat and vermiculite (7:3, v/v) in trays in a growth chamber. When the first true leaf fully expanded, seedlings were transplanted into plastic pots (15cm diameter and 15cm deep, one seedling per pot) containing the same medium and were watered daily with Hoagland nutrient solution. The tomato plants were grown in the growth chamber at 25/20°C, 600 μE m^-2^ s^-1^ light with 12h light/12h darkness.

### Generation of WRKY transgene expression constructs and transgenic lines

The full-length coding sequences for various WRKY genes were first PCR amplified using the gene-specific primers listed in Supplementary Table S1. For generating transgenic over-expression lines, the WRKY coding sequences were inserted behind the *CaMV 35S* promoter in a plant transformation vector (POCA30 or pFGC5941). For generating WRKY transgenes driven by the *AtWRKY33* or *AtWRKY25* promoter, the ~1.5kb DNA fragments upstream of the translation start codons for the WRKY genes were first PCR-amplified using primers listed in Supplementary Table S1 and were subcloned to replace the CaMV *35S* promoter in a plant expression vector. The expression constructs for the various WRKY genes were transformed into Arabidopsis *atwrky33* mutant plants using the *Agrobacterium*-mediated floral dip procedure (Clough and Bent, 1998). F2 progeny from two independent lines expressing high levels of a transgene were used for analysis of *Botrytis* resistance and heat tolerance (Supplementary Fig. S1). Transformants were identified for resistance to Basta or kanamycin. Transgenic plants overexpressing the transformed WRKY transgene were identified by northern blotting.

### VIGS constructs and Agrobacterium-mediated virus infection

Two ~520bp *SlWRKY33A* and *SlWRKY33B* coding sequences were PCR-amplified using gene-specific primers (*SlWRKY33A*: 5ʹ-ATCGAATTCCCATTGCAGTCTTGTATCT-3ʹ and 5ʹ-ATCCTCGAGTGTTTTGTGGGCTCTTGACA-3ʹ; *SlWRKY33B*: 5ʹ-ATCGAATTCCATCTGGAAGCAACAACA 3ʹ and 5ʹ-ATCCTCGAGCATGAAAACTCAGTTCCACCT-3ʹ). The PCR products were digested with EcoRI and SacI and cloned into the same sites of pTRV2 VIGS vector (Liu *et al.*, 2002). The resulted silencing vectors were transformed into *Agrobacterium* strain GV3101 and silencing of the targeted genes was determined by qRT-PCR using the primers listed in Supplementary Table S2. *Agrobacterium*-mediated virus infection was performed as previously described (Liu *et al.*, 2002). Plants were then kept at 23/21ºC under 120 μE m^-2^ s^-1^ light for 30 d before further analysis.

### Total RNA extraction and quantitative RT-PCR

Total RNA was isolated from Arabidopsis or tomato leaves using the Trizol reagent (Sangon, China), according to the supplier’s instruction. Genomic DNA was removed with the RNeasy Mini Kit (Qiagen, Germany). Total RNA (1mg) was reverse-transcribed using ReverTra Ace qRT-PCR Kit (Toyobo, Japan), following the manufacturer’s instructions. Quantitative RT-PCR was performed with an iCycleriQ Multicolor Real-Time PCR Detection System (Bio-Rad, USA). PCRs were performed using the SYBR Green PCR Master Mix (Applied Biosystems) and gene-specific primers listed in Supplementary Table S2. The PCR conditions consisted of denaturation at 95°C for 3min, followed by 40 cycles of denaturation at 95°C for 30 s, annealing at 58°C for 30 s and extension at 72°C for 30 s. Relative gene expression was calculated as previously described (Livak and Schmittgen, 2001) using Arabidopsis *Actin2* gene or tomato *Actin* gene as internal control.

### Analysis of disease resistance and heat tolerance

Culture and inoculation of *B. cinerea* were performed as previously described (Zheng *et al.*, 2006). Biomass of the fungal pathogen was quantified by qRT-PCR from inoculated plants for the *Botrytis ActA* gene transcript levels. For testing heat tolerance, Arabidopsis plants were placed in a 22ºC or 45ºC growth chamber for 10h and then immediately analysed for electrolyte leakage (EL) or Fv/Fm as previously described (Huang *et al.*, 2010), or moved to room temperature for 3–5 d recovery for observation of heat stress symptoms.

### Accession numbers

Sequence data for the genes described in this study can be found in the GenBank/EMBL data libraries under the accession numbers shown in parentheses: *ATWRKY25* (At2g30250), *ATWRKY33* (At2g38470), *ATActin2* (AT3g18780), *SlWRKY33A* (Sl06g066370), *SlWRKY33B* (Sl09g014990), *SlActin* (Sl11g005330).

## Results

### The C-terminal domains (CTDs) of AtWRKY33 and its close homologues

**Fig. 1.**
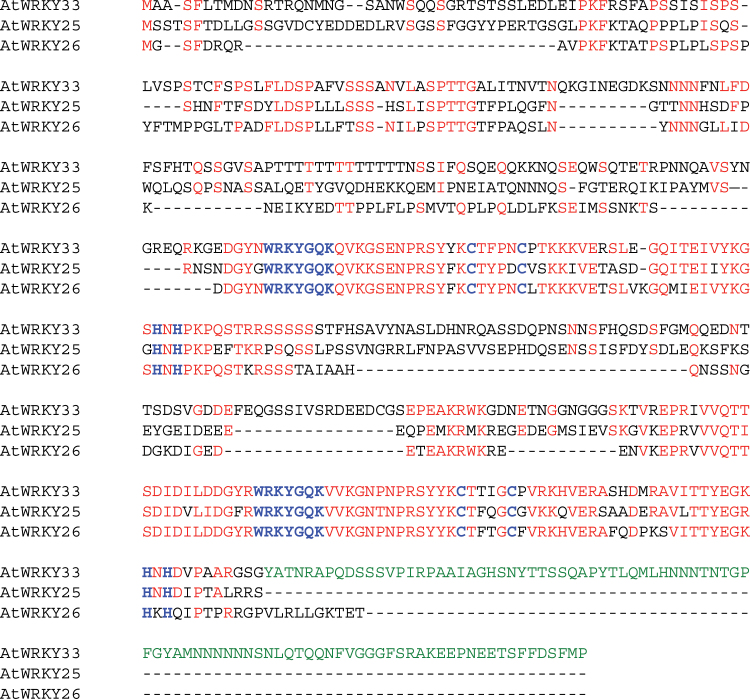
Protein sequence comparison of AtWRKY33, AtWRKY25 and AtWRKY26. Amino acid residues of AtWRKY25 or AtWRKY26 identical to those of AtWRKY33 are in red. The highly conserved WRKYGQK sequences and the residues forming the C_2_H_2_ zinc fingers are in blue. The amino acid residues on the C-terminal side of the C-terminal WRKY domain of AtWRKY33, which are absent in AtWRKY25 or AtWRKY26, are in green.

In Arabidopsis, AtWRKY25 and AtWRKY26 are the two closest homologues of AtWRKY33 and, as a result, have been studied with AtWRKY33 for possible redundant roles in plant responses to biotic and abiotic stresses (Petersen *et al.*, 2000; Li *et al.*, 2011). However, the strong phenotypes of the *atwrky33* mutants in resistance to necrotrophic pathogens and pathogen-induced phytoalexin biosynthesis were not observed in the *atwrky25* or *atwrky26* mutants. To study the possible structural basis for the diversified biological functions among the three closely related WRKY transcription factors, we first compared their structures through amino acid alignment (Fig. 1). As Group I WRKY proteins, AtWRKY33, AtWRKY25 and AtWRKY26 all contain two WRKY domains with highly conserved amino acid sequences (Fig. 1). For the N-terminal domains, all three proteins contain a highly conserved motif with clustered proline-directed serine (SP cluster) as potential phosphorylation sites, which are present even in the WRKY protein of the unicellular green algae *Chlamydomonas* (Ishihama and Yoshioka, 2012). However, sequence alignment with AtWRKY33 revealed deletions of four relatively long segments of ~10–30 amino acid (aa) residues for AtWRKY26 and three short segments of 5–9 aa for AtWRKY25 (Fig. 1). Likewise, the intervening sequence between the two WRKY domains in AtWRKY26 is substantially shortened due to absence of three segments of ~11–32 aa while AtWRKY25 has only a single segment of 16 aa absent in the intervening sequence when aligned to AtWRKY33 (Fig. 1). The most striking difference between AtWRKY33 and its two close homologues is at the C-terminus. While AtWRKY33 contains a segment of ~100 aa on the C-terminal side of the C-terminal WRKY domain, neither AtWRKY25 nor AtWRKY26 contains such an extended CTD (Fig. 1). Thus, although AtWRKY25, AtWRKY26 and AtWRKY33 are often described as close structural homologues, their structural diversity is still substantial, particularly at the C-terminus.

### The critical roles of AtWRKY33 CTD and its native promoter for its full activity

**Fig. 2.**
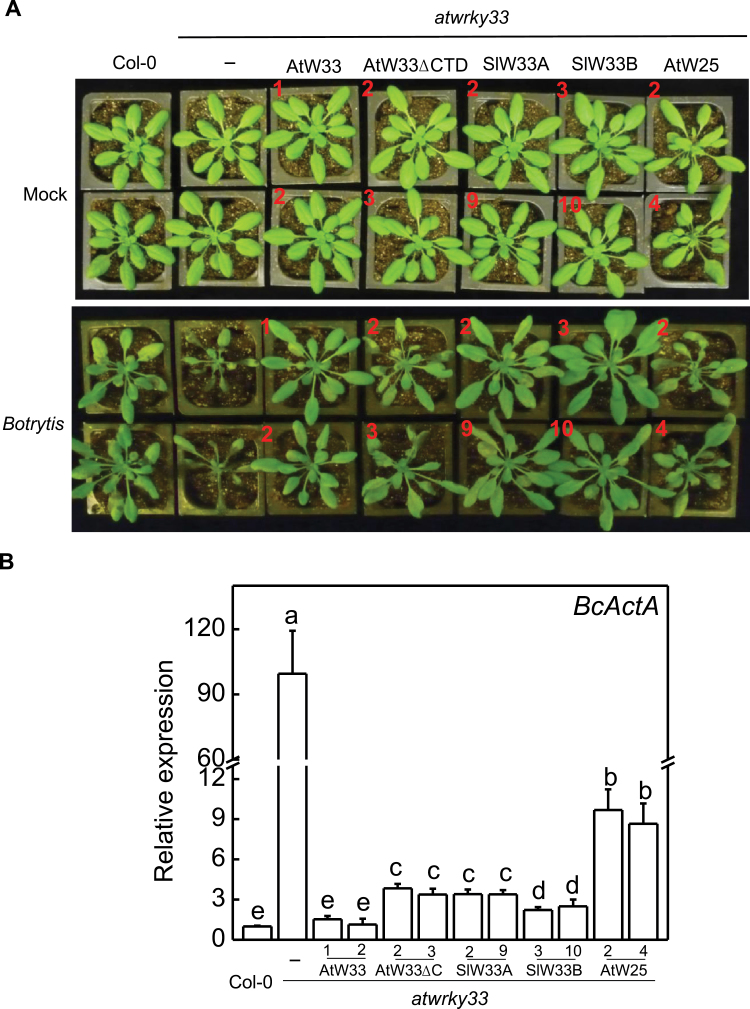
Complementation of *atwrky33* mutant plants for *Botrytis* resistance by *AtWRKY33* (AtW33), *AtWRKY33DCTD* (AtW33DCTD), *SIWRKY33A* (SlW33A), *SIWRKY33B* (SlW33B) and *AtWRKY25* (AtW25) driven by the CaMV *35S* promoter. (A) Disease symptom development. Col-0 wild type, *atwrky33* and transgenic *atwrky33* lines constitutively expressing the various WRKY transgenes were sprayed inoculated with buffer (mock) or spores of *Botrytis* (Botrytis). The pictures of representative plants from two independent lines (indicated by numbers in red) for each transgene were taken at 4 dpi. (B) The expression of the *Botrytis ActinA* gene in spray-inoculated plants at 4 dpi. Total RNA of wild type, mutant and two independent lines of each transgene was isolated from leaf samples, and transcript levels were determined using qRT-PCR with Arabidopsis *Actin2* gene as internal control. Error bars indicate SE (*n*=3). According to Duncan’s multiple range test (*P*=0.05), means of lesion areas do not differ significantly if they are indicated with the same letter.

**Fig. 3.**
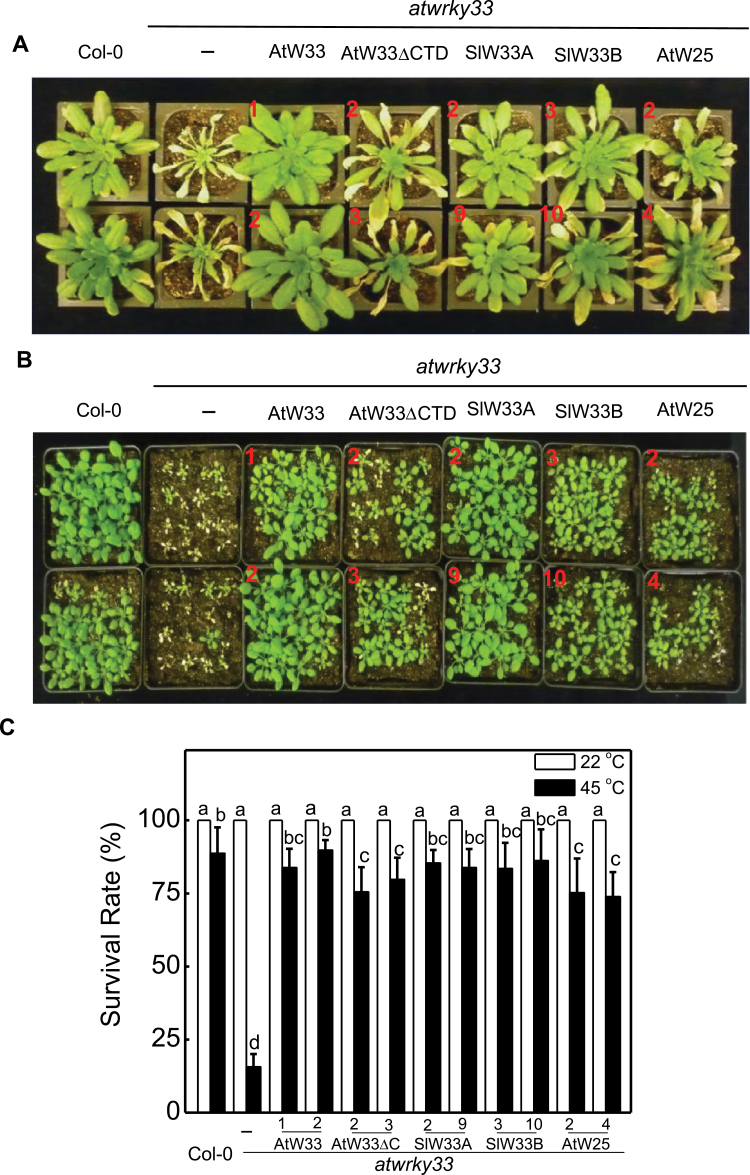
Complementation of *atwrky33* mutant plants for heat tolerance by *AtWRKY33* (AtW33), *AtWRKY33DCTD* (AtW33DCTD), *SIWRKY33A* (SlW33A), *SIWRKY33B* (SlW33B) and *AtWRKY25* (AtW25) driven by the CaMV *35S* promoter. (A) Assays of heat tolerance of mature plants. Col-0 wild type, *atwrky33* and transgenic *atwrky33* lines constitutively expressing the various WRKY transgenes were placed in a 45°C growth chamber for 10h. The heat-treated plants were then moved to a 22°C growth chamber for recovery. The pictures of representative plants from two independent lines (indicated by numbers in red) for each transgene were taken 3 d after the heat treatment. (B) Assays of heat tolerance of young seedlings. Two-week-old Col-0 wild type, *atwrky33* and transgenic *atwrky33* lines constitutive expressing the various WRKY transgenes were placed in a 45°C growth chamber for 10h. The heat-treated plants were then moved to a 22°C growth chamber for recovery. The pictures of seedlings from two independent transgenic lines (indicated by numbers in red) for each transgene were taken 3 d after the heat treatment. (C) Survival rates of heat-stressed young seedlings. Means and SE were calculated from average survival rates determined from three experiments with 25 plants per experiment for wild type, mutant and two independent lines of each transgene. Error bars indicate SE (*n*=3). According to Duncan’s multiple range test (*P*=0.05), means of survival rates do not differ significantly if they are indicated with the same letter.

For functional analysis of AtWRKY33 CTD, we determined whether its removal compromised the ability of AtWRKY33 to restore disease resistance and stress tolerance to the *atwrky33* mutants. For this purpose, we generated a mutant AtWRKY33 protein in which the C-terminal 94 aa of AtWRKY33 was removed (AtWRKY33DCTD). Both *AtWRKY33* and *AtWRKY33DCTD* gene were inserted into a plant expression vector behind the constitutive CaMV *35S* promoter. For comparison, the coding sequence for AtWRKY25, which does not contain an extended CTD, was also placed into the same vector behind the strong promoter. All these three constructs were transformed into the *atwrky33* mutant plants and transgenic plants constitutively expressing the transgenes were identified (Supplementary Fig. S1) and the progeny of two independent transgenic lines were tested for disease resistance to *Botrytis* based on both disease symptom development and accumulation of transcripts for *Botrytis ActinA* genes as an indicator for fungal growth on inoculated plants. As expected, the *atwrky33* mutant was compromised in the disease resistance as indicated from enhanced disease symptoms after *Botrytis* infection (Fig. 2). The *atwrky33* mutant plants were also compromised in heat tolerance at 45ºC as indicated from symptoms of mature plant development and reduced seedling survival rate after the heat shock (Fig. 3). Transformation of *atwrky33* with the wild-type *AtWRKY33* gene completely restored the *Botrytis* resistance and heat tolerance to the mutant (Figs 2, 3). Interestingly, in the transgenic *atwrky33* mutant plants expressing *AtWRKY25* or *AtWRKY33DCTD* driven by the strong CaMV *35S* promoter, resistance to *Botrytis* was also largely restored as both disease symptoms and fungal growth were comparable to those in wild-type plants (Figs 2, 3). Both *AtWRKY25* and *AtWRKY33DCTD* driven by the strong CaMV *35S* promoter also restored the heat tolerance to the *atwrky33* mutant plants when assayed using mature plants (Fig. 3A). The two genes also restored the heat tolerance to the *atwrky33* mutant plants based on the increased survival rates of the transgenic plants, although growth of the surviving plants was significantly reduced during recovery following heat stress (Fig. 3B, C). Taken together, both *AtWRKY25* and *AtWRKY33DCTD* genes driven by the strong CaMV *35S* promoter largely restored both the *Botrytis* resistance and heat tolerance to the *atwrky33* mutant plants.

**Fig. 4.**
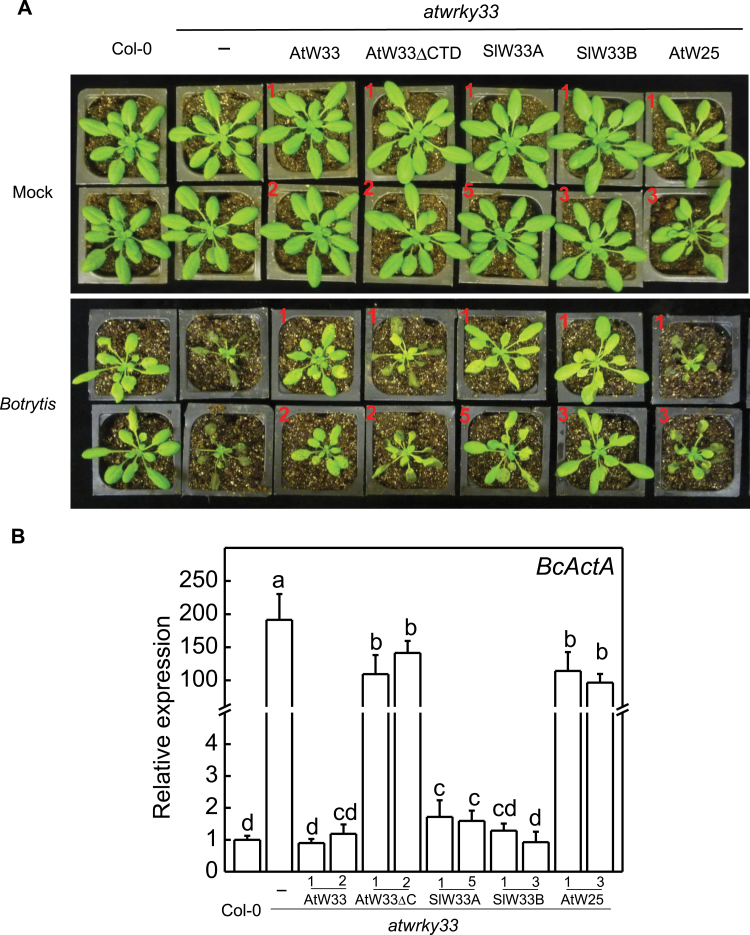
Complementation of *atwrky33* mutant plants for *Botrytis* resistance by *AtWRKY33* (AtW33), *AtWRKY33DCTD* (AtW33DCTD), *SIWRKY33A* (SlW33A), *SIWRKY33B* (SlW33B) and *AtWRKY25* (AtW25) driven by the *AtWRKY33* promoter. (A) Disease symptom development. Col-0 wild type, *atwrky33* and transgenic *atwrky33* lines constitutively expressing the various WRKY transgenes were spray-inoculated with buffer (mock) or spores of *Botrytis* (Botrytis). The pictures of representative plants from two independent lines (indicated by numbers in red) for each transgene were taken at 4 dpi. (B) The expression of the *Botrytis ActinA* gene in spray-inoculated plants at 4 dpi. Total RNA of wild type, mutant and two independent lines of each transgene was isolated from leaf samples, and transcript levels were determined using qRT-PCR with Arabidopsis *Actin2* gene as internal control. Error bars indicate SE (*n*=3). According to Duncan’s multiple range test (*P*=0.05), means of survival rates do not differ significantly if they are indicated with the same letter.

Since the *atwrky33* mutant plants express the *AtWRKY25* endogenous gene despite compromised disease resistance and heat sensitivity, we reasoned that the ability of the *AtWRKY25* transgene, and perhaps also the *AtWRKY33DCTD* transgene, in restoring both disease resistance and heat tolerance to *atwrky33* resulted from their strong constitutive expression conferred by the CaMV *35S* promoter. To test this, we placed *AtWRKY33*, *AtWRKY25* and *AtWRKY33DCTD* genes under the native *AtWRKY33* promoter and transformed them into the *atwrky33* mutant plants. Transgenic lines were identified and their stable progeny were again tested for disease resistance and heat tolerance. As shown in Fig. 4, at three days post *Botrytis* inoculation (dpi), wild-type Col-0 plants displayed few symptoms and supported little growth of the fungal pathogen based on qRT-PCR of the fungal *ActinA* transcripts. As expected the *atwrky33* mutant plants displayed severe tissue maceration (Fig. 4A) and prolific fungal growth (Fig. 4B). The resistance to the fungal pathogen was fully restored in *atwrky33* by the *ATWRKY33* gene driven by its native promoter (Fig. 4). In contrast, in transgenic at*wrky33* mutant plants expressing *AtWRKY25* or *AtWRKY33DCTD* driven by the *ATWRKY33* promoter, both the severe disease symptoms and fungal growth were similar to those in the *atwrky33* mutants (Fig. 4). Thus, both *AtWRKY25* and *AtWRKY33DCTD* driven by the *ATWRKY33* gene promoter were ineffective in restoring the *Botrytis* resistance to the *atwrky33* mutants.

**Fig. 5.**
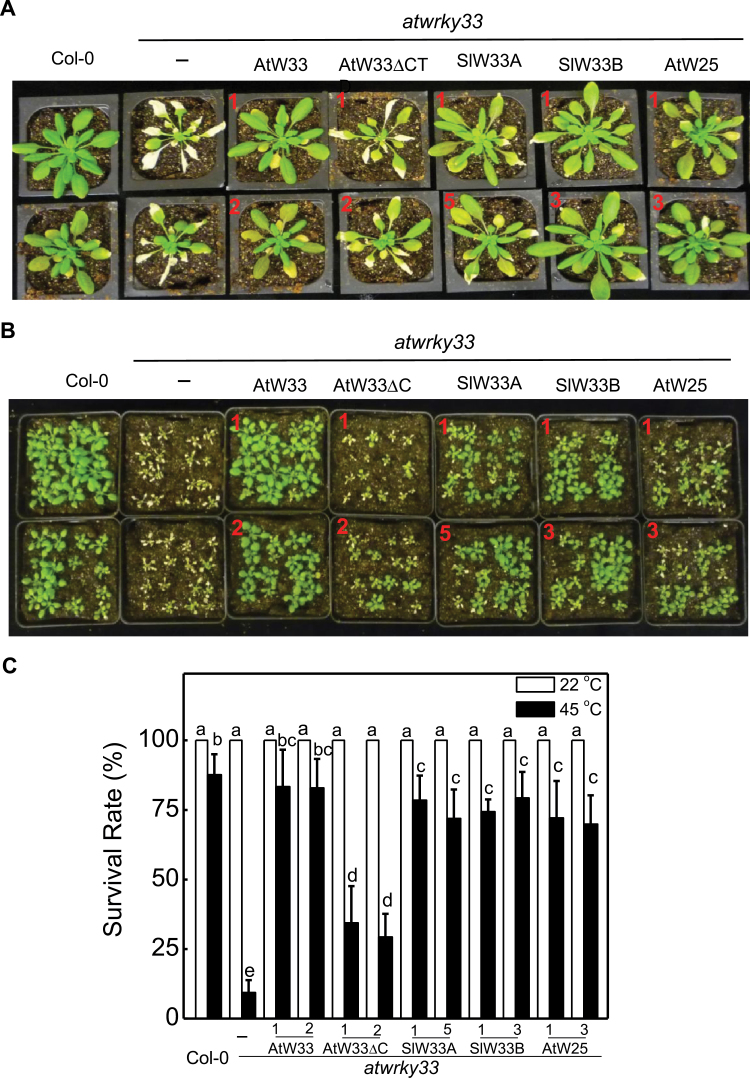
Complementation of *atwrky33* mutant plants for heat tolerance by *AtWRKY33* (AtW33), *AtWRKY33DCTD* (AtW33DCTD), *SIWRKY33A* (SlW33A), *SIWRKY33B* (SlW33b) and *AtWRKY25* (AtW25) driven by the *AtWRKY33* promoter. (A) Assays of heat tolerance of mature plants. Col-0 wild type, *atwrky33* and transgenic *atwrky33* lines constitutively expressing the various WRKY transgenes were placed in a 45°C growth chamber for 10h. The heat-treated plants were then moved to a 22°C growth chamber for recovery. The pictures of representative plants from two independent lines (indicated by numbers in red) for each transgene were taken 3 d after the heat treatment. (B) Assays of heat tolerance of young seedlings. Two-week-old Col-0 wild type, *atwrky33* and transgenic *atwrky33* lines constitutively expressing the various WRKY transgenes were placed in a 45°C growth chamber for 10h. The heat-treated plants were then moved to a 22°C growth chamber for recovery. The pictures of seedlings from two independent lines (indicated by numbers in red) for each transgene were taken 3 d after the heat treatment. (C) Survival rates of heat-stressed young seedlings. Means and SE were calculated from average survival rates determined from three experiments with 25 plants per experiment for wild type, mutant and two independent lines of each transgene. Error bars indicate SE (*n*=3). According to Duncan’s multiple range test (*P*=0.05), means of survival rates do not differ significantly if they are indicated with the same letter.

We also tested *AtWRKY25* or *AtWRKY33DCTD* driven by the *ATWRKY33* gene promoter for their ability to restore heat tolerance to the *atwrky33* mutant. In mature plants, expression of *AtWRKY33* or *AtWRKY25* driven by the *ATWRKY33* promoter almost completely eliminated extensive heat-induced tissue damage observed in the *atwrky33* mutant plants (Fig. 5A). Expression of *AtWRKY33DCTD* driven by the *ATWRKY33* promoter also significantly reduced but not completely eliminated heat-induced leaf tissue damage in *atwrky33* (Fig. 5A). In young seedlings, expression of *AtWRKY33* driven by its native promoter again completely complemented the *atwrky33* mutant for the high survival rates and growth of the surviving plants during recovery following heat stress (Fig. 5B, C). The survival rates and growth of the surviving plants of *atwrky33* were also partially recovered by *AtWRKY25* and, to a less extent, by *AtWRKY33DCTD* when driven by the *AtWRKY33* promoter (Fig. 5B, C). Thus, when expressed under control of the *AtWRKY33* promoter, *AtWRKY33DCTD* is partially effective while *AtWRKY25* was almost as effective as *AtWRKY33* in restoring the heat tolerance to the *atwrky33* mutant.

**Fig. 6.**
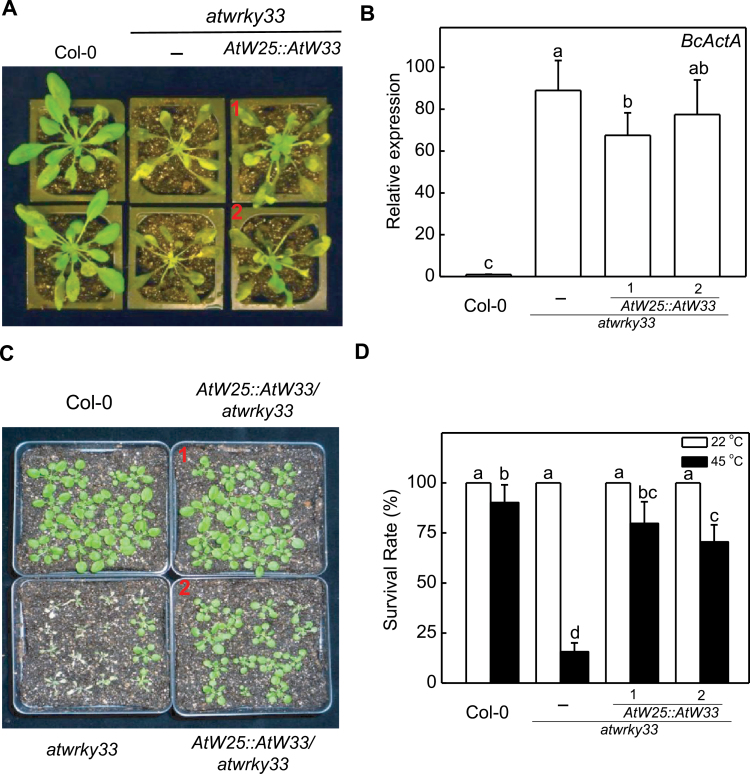
Complementation of *atwrky33* mutant for *Botrytis* resistance and heat tolerance by *AtWRKY33* driven by the *AtWRKY25* promoter. (A) Disease symptom development. Col-0 wild type, *atwrky33* and two independent transgenic *atwrky33* lines constitutively expressing *AtWRKY33* driven by the *AtWRKY25* promoter were spray-inoculated with buffer (mock) or spores of *Botrytis* (Botrytis). The pictures of representative plants from two independent lines (indicated by numbers in red) were taken at 4 dpi. (B) The expression of the *Botrytis ActinA* gene in spray-inoculated plants at 4 dpi. (C) Assays of heat tolerance of young seedlings. Two-week-old Col-0 wild type, *atwrky33* and transgenic *atwrky33* lines constitutively expressing *AtWRKY33* driven by the *AtWRKY25* promoter were placed in a 45°C growth chamber for 10h. The heat-treated plants were then moved to a 22°C growth chamber for recovery. The pictures of seedlings from two independent transgenic lines (indicated by numbers in red) were taken 3 d after the heat treatment. (D) Survival rates of heat-stressed young seedlings. Means and SE were calculated from average survival rates determined from three experiments with 25 plants per experiment for wild type, mutant and two independent lines of each transgene. Error bars indicate SE (*n*=3). According to Duncan’s multiple range test (*P*=0.05), means of survival rates do not differ significantly if they are indicated with the same letter.

To further determine the role of gene promoters, we isolated the *AtWRKY25* gene promoter and used it to drive the expression of *AtWRKY33*. As shown in Fig. 6A, B, in the transgenic at*wrky33* mutant expressing *AtWRKY33* driven by the *ATWRKY25* gene promoter, both the severe disease symptoms and prolific fungal growth were comparable to those in the *atwrky33* mutants. On the other hand, compromised heat tolerance of the *atwrky33* mutant was substantially improved by *AtWRKY33* driven by the *AtWRKY25* promoter (Fig. 6C, D). These data indicated that not only the AtWRKY33 structure, exemplified by its CTD, but also its promoter are important for the full activity of AtWRKY33, particularly in plant resistance to *Botrytis*.

**Fig. 7.**
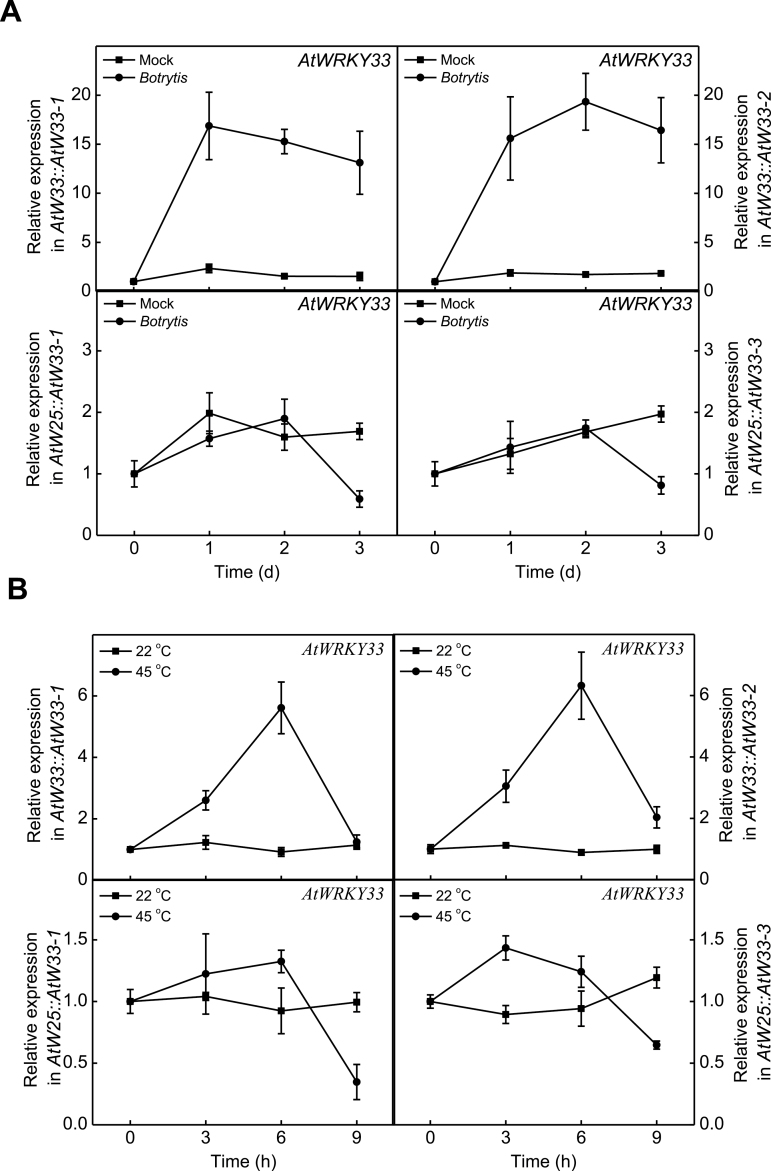
AtWRKY33 transgene expression conferred by the *AtWRKY33* or *AtWRKY25* gene promoter in response to *Botrytis* infection or heat stress. (A) *Botrytis*-induced and (B) heat-induced expression of *AtWRKY33* gene in the transgenic *atwrky33* mutant plants harbouring the *AtWRKY33* transgene gene driven by the promoter of *AtWRKY33* (*AtW33::AtW33*) or *AtWRKY25* (*AtW25::AtW33*). Total RNA of wild type, mutant and two independent transgenic *atwrky33* lines constitutively expressing *AtWRKY33* driven by the *AtWRKY33* or *AtWRKY25* promoter was isolated from leaf samples at the indicated day post inoculation of *Botrytis* (dpi), and transcript levels were determined using qRT-PCR with Arabidopsis *Actin2* gene as internal control. Error bars indicate SE (*n*=3).

Both *AtWRKY33* and *AtWRKY25* are responsive to biotic and abiotic stimuli (Dong *et al.*, 2003; Zheng *et al.*, 2006; Lippok *et al.*, 2007; Zheng *et al.*, 2007; Jiang and Deyholos, 2009; Li *et al.*, 2009, 2011) and, therefore, it is unclear how the AtWRKY33 driven by the two pathogen/stress-responsive promoters displayed such a major difference in conferring disease resistance to *Botrytis* and heat tolerance (Fig. 5). To address the question, we compared the expression of *AtWRKY33* transgene under control of the two promoters in the transgenic *atwrky33* mutant plants in response to *Botrytis* infection or heat stress. As shown in Fig. 7A, *AtWRKY33* transgene under control of the *AtWRKY33* promoter was rapidly induced in *Botrytis*-inoculated plants, however, *AtWRKY33* transgene under control of the *AtWRKY25* promoter was not significantly induced in *Botrytis*-inoculated plants (Fig. 7A). Likewise, we observed higher levels of *AtWRKY33* transgene transcripts when driven by the *AtWRKY33* promoter in heat-treated *atwrky33* mutant plants compared with controls, but the expressions of *AtWRKY33* were not induced when driven by the *AtWRKY25* promoter under heat stress (Fig. 7B).

### Identification of close WRKY33 homologues in other plants

**Fig. 8.**
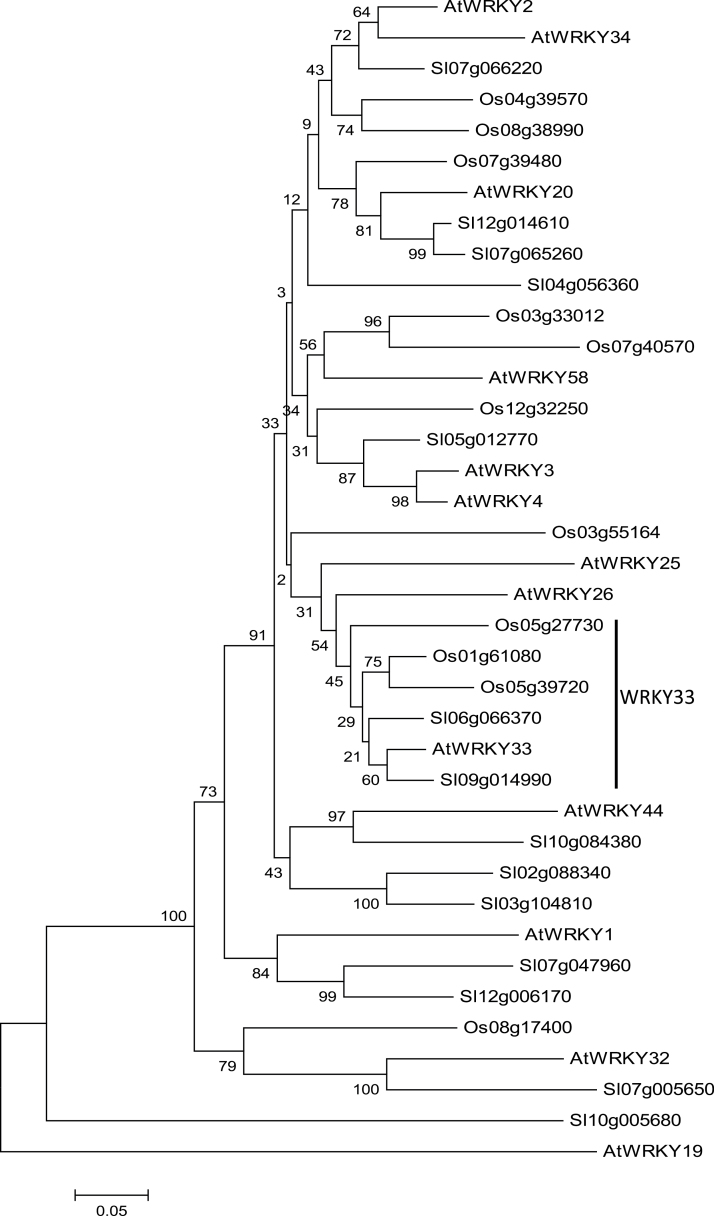
Phylogenetic tree of group I WRKY proteins from Arabidopsis, tomato, and rice. The tree was inferred from the C-terminal portion of the proteins starting from the highly conserved WRKYGQK sequence of the N-terminal WRKY domains using the neighbour-joining method. Phylogenetic analysis was conducted in MEGA5. Bootstrap values from 1000 replicates were used to assess the robustness of the tree. The WRKY proteins closely related to AtWRKY33 are indicated by a vertical line.

Given the broad and critical roles of AtWRKY33, we examined whether there are evolutionarily conserved WRKY33 homologues in important crop plants using phylogenetic and sequence analysis. Previous structural analyses of WRKY proteins for classification of the superfamily of transcription factors have often focused on the numbers and structures of conserved WRKY domains (Eulgem *et al.*, 2000; Zhang and Wang, 2005). In light of the critical role of AtWRKY33 CTD, we performed a phylogenetic analysis of Group I WRKY proteins from Arabidopsis, tomato (a dicot) and rice (a monocot) using the C-terminal portion of the proteins starting from the highly conversed WRKYGQK sequence of their N-terminal WRKY domains. Based on the resultant phylogenetic tree, AtWRKY33 consistently forms a close cluster with two tomato WRKY proteins (Sl09g014990 and Sl06g066370) and three rice WRKY proteins (Os01g61080, Os05g39720 and Os05g27730) (Fig. 8). Thus, the phylogenetic analysis indicated that AtWRKY33 is structurally unique among the 12 Group I Arabidopsis WRKY proteins but has close structural homologues in both monocot and dicot plants. Sl09g014990 and Sl06g066370, which are clustered with AtWRKY33 in the phylogenetic analysis, are the previously identified SlWRKY33A and SlWRKY33B, respectively (Zhou *et al.*, 2014). When the sequences of AtWRKY33, SlWRKY33A and SlWRKY33B were compared, all contain two WRKY domains with highly conserved amino acid sequences. High sequence similarities are also found in the N-terminal domains including the highly conserved SP clusters as putative MAPK phosphorylation sites and the intervening sequences between the two WRKY domains (Zhou *et al.*, 2014). Most notably, both SlWRKY33A and SlWRKY33B contain a segment of about 100 aa on the C-terminal side of the second WRKY domain with substantial sequence homology with AtWRKY33 CTD (Zhou *et al.*, 2014).

### Stress-induced expression of tomato *SlWRKY33* genes

**Fig. 9.**
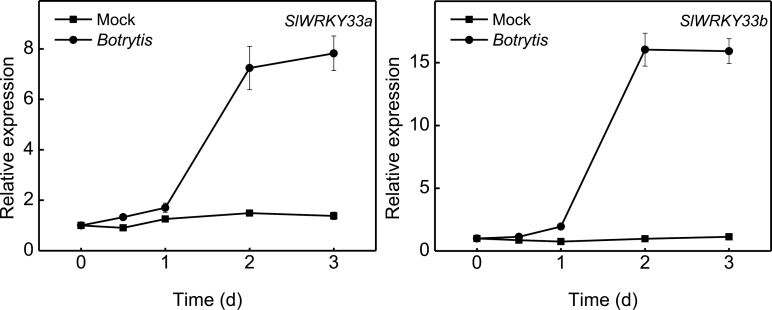
*Botrytis*-induced expression of tomato *SlWRKY33A* and *SlWRKY33B*. Tomato leaves were drop-inoculated with buffer (mock) or *Botrytis* spores and leaf tissues were collected at indicated times for total RNA isolation and determination of the gene transcript levels using qRT-PCR with tomato *Actin* gene as internal control. Error bars indicate SE (*n*=3).

To determine whether structurally related WRKY33 homologues from different plants are also functionally conserved, we chose to functionally analyse tomato SlWRKY33A and SlWRKY33B because both *Botrytis* resistance and heat tolerance can be tested in tomato, making the direct comparison possible with Arabidopsis. As a first step to determine the biological roles of tomato *SlWRKY33A* and *SlWRKY33B*, we analysed their expression in response to *Botrytis* infection and heat treatment. Tomato plants were inoculated with *Botrytis* and analysed for expression of *SlWRKY33A* and *SlWRKY33B* using qRT-PCR. For both genes, increased levels of transcripts were detected as early as 1 dpi but the major induction was observed at 2 and 3 dpi (Fig. 9). Heat-induced expression of *SlWRKY33A* and *SlWRKY33B* has been analysed by comparing their transcript levels in tomato plants placed in a 22°C or 45°C growth chamber (Zhou *et al.*, 2014). The transcript levels of *SlWRKY33A* and *SlWRKY33B* remained constantly low at 22°C but were elevated with similar kinetics at 45°C (Zhou *et al.*, 2014). Thus expression of the two tomato *WRKY33* genes was induced by both *Botrytis* infection and heat stress.

### Compromised disease resistance and heat tolerance in *SlWRKY33*-silenced tomato

To determine directly the roles of tomato *SlWRKY33* genes, we used virus-induced gene silencing (VIGS) to assess the impact of their down-regulated expression on tomato disease resistance and heat tolerance. *SlWRKY33*-specific DNA fragments were cloned into the pTRV vector and *Agrobacterium* cells harbouring the VIGS vectors were infiltrated into tomato leaves (Zhou *et al.*, 2014). We used qRT-PCR to compare the transcript levels for *SlWRKY33A* and *SlWRKY33B* in tomato plants infiltrated with the TRV empty vector or with the TRV-*SlWRKY33A* or TRV-*SlWRKY33B* silencing vector. As shown in Supplementary Fig. S2, basal expression of *SlWRKY33A* or *SlWRKY33B* was observed in the tomato plants infiltrated with the TRV empty vector. By contrast, infiltration with either TRV-*SlWRKY33A* or TRV-*SlWRKY33B* silencing vector resulted in approximately 4–6-fold reduction in the transcript levels for both TRV-*SlWRKY33A* and TRV-*SlWRKY33B* (Supplementary Fig. S2). The cross-silencing likely resulted from the high sequence homology between the two genes, which share more than 75% nucleotide sequence identify. The tomato plants silenced for both *SlWRKY33A* and *SlWRKY33B* were normal in growth and development and displayed no detectable morphological phenotype.

**Fig. 10.**
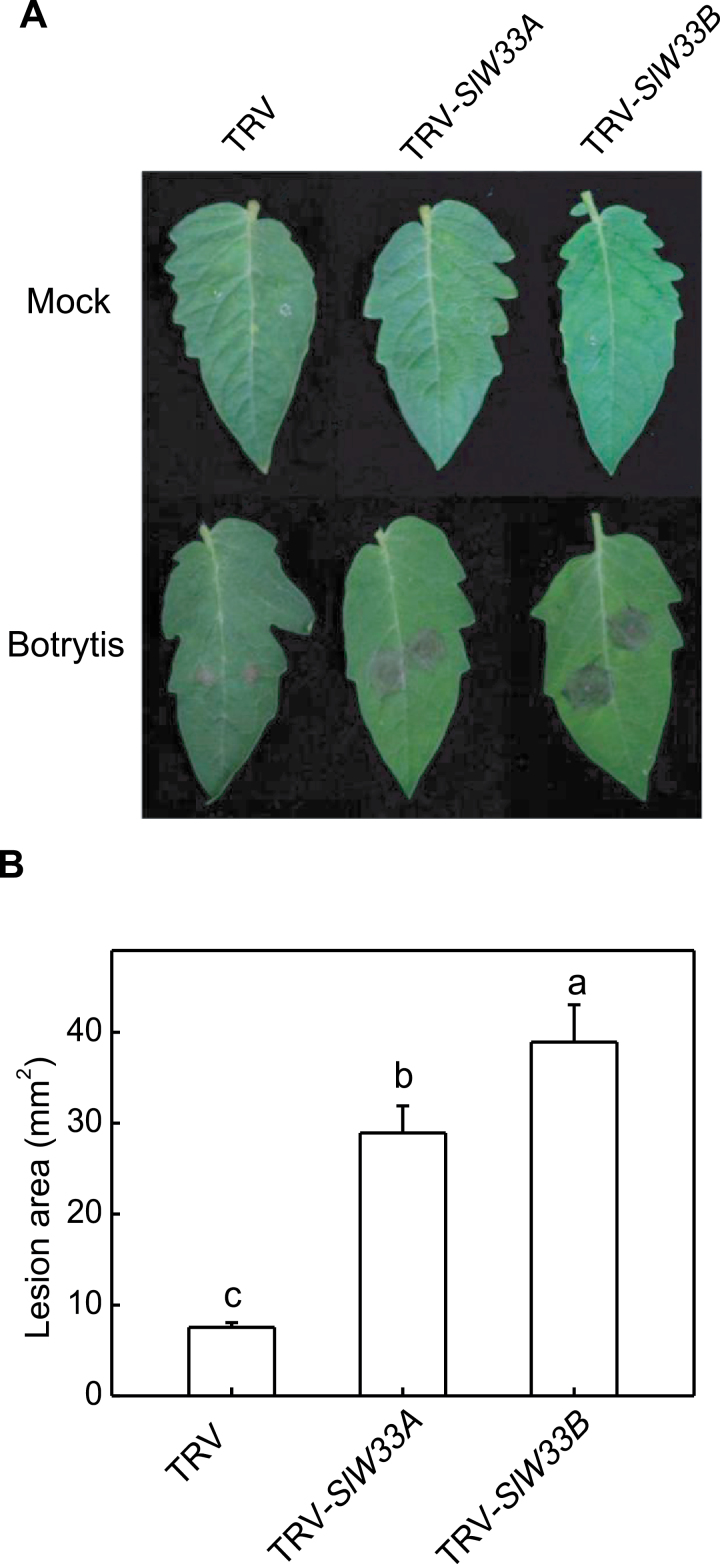
Functional analysis of SlWRKY33A and SlWRKY33B in tomato resistance to *Botrytis* using TRV-mediated gene silencing. (A) *Botrytis* symptom development. Tomato plants infiltrated with *Agrobacterium* cells harbouring the empty TVR vector or the silencing TRV-SlWRKY33A (TRV-SlW33a) or TRV-SlWRKY33B (TRV-SlW33b) vector. The terminal leaflets of the fourth leaves of *Agrobacterium*-infiltrated tomato plants were drop-inoculated with *Botrytis* spores. The picture was taken at 4 dpi. (B) *Botrytis* lesion size. The lesion areas were determined at 4 dpi. Means and SE were calculated from average lesion areas determined from three experiments with 10 leaves per experiment for each genotype. Error bars indicate SE (*n*=3). According to Duncan’s multiple range test (*P*=0.05), means of lesion areas do not differ significantly if they are indicated with the same letter.

When leaves of TRV-infiltrated tomato plants were drop-inoculated with *Botrytis* spores, the disease lesions were largely restricted to the inoculation sites (Fig. 10). In these leaves, expression of both *SlWRKY33A* and *SlWRKY33*B was greatly induced (Supplementary Fig. S2). In the leaves of pTRV-*SlWRKY33*-infiltrated tomato plants, drop inoculation with the fungal pathogen led to increased lesion expansion indicated from the 2–3-fold increase in the lesion areas relative to those of the TRV control plants (Fig. 10). The increased susceptibility in the pTRV-*SlWRKY33*-infiltrated tomato plants was associated with 5–7-fold reduction of *Botrytis*-induced transcript levels of *SlWRKY33* genes in the plants relative to those in TRV-infiltrated tomato plants (Supplementary Fig. S2). The effects of silencing of *SlWRKY33* genes on tomato heat tolerance were also been examined. Based on comprehensive analysis of symptoms of leaf dehydration, compromised capacity of PSII photochemistry and membrane integrity at 45°C, it was apparent that *SlWRKY33*-silenced plants were more severely compromised in heat tolerance than control plants (Zhou *et al.*, 2014). As with *Botrytis* resistance, compromised heat tolerance of the pTRV-SlWRKY33-infiltrated tomato plants was associated with strong reduction in heat-induced transcript levels for both *SlWRKY33A* and *SlWRKY33B* (Zhou *et al.*, 2014).

### Complementation of *atwrky33* mutants by tomato *SlWRKY33* genes

To further establish that SlWRKY33A and SlWRKY33B are orthologues of AtWRKY33, we performed complementation experiments in the *atwrky33* mutant plants. Full-length *SlWRKY33A* and *SlWRKY33B* coding sequences were cloned into a plant transformation vector behind the CaMV *35S* promoter or the *AtWRKY33* gene promoter and transformed into the *atwrky33* mutant plants. Positive transformants expressing the transgenes were identified by northern blotting and qRT-PCR (Supplementary Figs S1, S3) and two independent transgenic lines were used in the present study. When driven by the *AtWRKY33* promoter, the transcript levels for the *SlWRKY33* transgenes were induced in the transgenic *atwrky33* mutant plants after *Botrytis* infection or heat treatment (Supplementary Fig. S3). Stable progeny from these transgenic lines were tested for resistance to *Botrytis* and heat tolerance. As shown in Figs 2–5, transformation of *atwrky33* with *SlWRKY33* genes driven by the strong CaMV *35S* promoter or the *AtWRKY33* gene promoter fully restored resistance to *Botrytis* and heat tolerance to the mutant plants. Thus, *SlWRKY33A* and *SlWRKY33B* fully complemented the *atwrky33* mutant plants.

## Discussion

The WRKY gene family has expanded from a single member in the green unicellular algae *C. reinhardtii* to more than 100 members in many land plants. The expansion of the WRKY family likely reflects complex adaptation mechanisms evolved in land plants and, consequently, elaborate regulation of a large number of plant genes under harsh environmental conditions including desiccation, UV radiation and attack of microbial pathogens in terrestrial habitats. However, almost all analysed WRKY proteins recognize the TTGACC/T W-box sequences by their highly conserved WRKY domains and it is unclear how the functional diversification of WRKY transcription factors is achieved structurally and evolutionarily during the expansion of the protein superfamily. AtWRKY33 is a member of the ancestral Group I WRKY proteins with broad biological roles and dynamic and complex regulation in plant responses to both biotic and abiotic stresses. In this study, we have conducted structural, functional and evolutionary analysis of WRKY33 homologues as an approach to gain insights into the evolution of plant WRKY transcription factors.

### What makes AtWRKY33 a highly important regulator of plant stress responses?

Arabidopsis AtWRKY25 and AtWRKY33 are two Group I WRKY proteins with extensive sequence homologue not only in the two highly conserved WRKY domains but also in their N-terminal domains and the sequence intervening the WRKY domains (Fig. 1). Genes encoding the two WRKY genes are also responsive to biotic and abiotic conditions (Dong *et al.*, 2003; Zheng *et al.*, 2006; Lippok *et al.*, 2007; Jiang and Deyholos, 2009; Li *et al.*, 2009, 2011). Both proteins contain clustered proline-directed serines (SP cluster) as potential phosphorylation sites of MAPKs (Fig. 1) and interact with similar VQ proteins (Lai *et al.*, 2011a; Cheng *et al.*, 2012), suggesting that they are both subject to regulation through protein phosphorylation and protein-protein interactions during plant defence and stress responses. Despite the similarities, the strong phenotypes of compromised resistance to necrotrophic pathogens in *atwrky33* mutants are not observed in *atwrky25* mutants (Zheng *et al.*, 2006). Since the extended CDT in AtWRKY33 is conspicuously absent in AtWRKY25 (Fig. 1), we first analysed whether the CTD is a critical structural element functionally distinguishing AtWRKY33 from AtWRKY25. Intriguingly, both AtWRKY25 and the CTD deletion mutant of AtWRKY33 (AtWRKY33DCTD) could restore, to great extents, *Botrytis* resistance when overexpressed under of the constitutive CaMV *35S* promoter (Fig. 2). However, if driven by the *AtWRKY33* promoter, neither *AtWRKY25* nor *AtWRKY33DCTD* was effective in complementing the *atwrky33* mutant for *Botrytis* resistance (Fig. 4). Furthermore, in the *atwrky33* mutant plants harbouring *AtWRKY33* driven by the *AtWRKY25* promoter, resistance to *Botrytis* was still severely compromised (Fig. 6). These results indicate that both the CTD and the promoter are important elements that distinguish AtWRKY33 from AtWRKY25 for the critical roles in plant resistance to necrotrophic pathogens. On the other hand, both its CTD and the promoter play a significant but not indispensible role of AtWRKY33 in plant heat tolerance since both *AtWRKY25* and *AtWRKY33DCTD* driven by the *AtWRKY33* promoter or *AtWRKY33* driven by the *AtWRKY25* promoter could substantially restore heat tolerance to the *atwrky33* mutant (Figs 5, 6B). This interpretation is consistent with the previous findings that AtWRKY33 and AtWRKY25 both play a significant role as indicated from partial functional redundancy in plant heat tolerance (Li *et al.*, 2009, 2011).

There are a number of mechanisms by which the structural and regulatory elements of AtWRKY33 can influence the regulatory activity and capacity of the transcription factor. There is no conserved domain in the ~100-amino acid CTD of AtWRKY33 and similar sequences are found only in plant WRKY33 homologues. As a structural motif immediately adjacent to the C-terminal DNA-binding WRKY domain, the CTD might enhance the regulatory activity of AtWRKY33 by positively modulating the DNA-binding activity or specificity of AtWRKY33. It could also positively regulate the stability and other properties of AtWRKY33 by mediating interaction of AtWRKY33 with other proteins. To test these possibilities, we have generated myc-tagged AtWRKY25, AtWRKY33 and AtWRKY33DCTD and transformed them into Arabidopsis under control of CaMV *35S* or the *AtWRKY33* promoter. However, comparative analysis of the transgenic plants using RNA and protein blotting indicated that the CTD of AtWRKY33 did not significantly affect protein stability (data not shown). We have also attempted to identify proteins interacting with the CTD of AtWRKY33 using yeast two-hybrid screening but failed to isolate any positive clones. Therefore, the specific properties of AtWRKY33 that are subject to the regulation by its CTD remain to be elucidated.

With regard to the promoter, the *AtWRKY33* promoter is apparently stronger and more responsive to *Botrytis* infection (Fig. 7). The *AtWRKY33* promoter contains a set of W boxes, which are critical for pathogen-induced *AtWRKY33* expression (Lippok *et al.*, 2007). It has been further shown recently that AtWRKY33 is activated upon phosphorylation by stress/pathogen-responsive MPK3 and MPK6 and activated AtWRKY33 activates its own expression through direct binding to its promoter (Mao *et al.*, 2011). The self-activation of *AtWRKY33* expression by MPK3/6-activated AtWRKY33 would generate a positive feedback mechanism for rapid and strong induction of *AtWRKY33* and its target genes. In addition, we have previously observed using GFP-tagged ATG8a as a marker that in *Botrytis*-infected wild-type plants, induced autophagosome formation was detectable in the lesion areas but high levels of induction were observed actually in areas surrounding the lesions and in distal areas in which no fungal mycelium was observed (Lai *et al.*, 2011b). In *atwrky33*, pathogen-induced autophagosome formation was still detectable in the lesion areas but absent in the surrounding areas (Lai *et al.*, 2011b). Therefore, induction of AtWRKY33-mediated defence genes takes place not only in infected plant cells but also in surrounding areas, leading to activation of plant defence mechanisms in a temporal and spatial pattern highly effective for containing pathogen proliferation.

Since both *AtWRKY33DCTD* and *AtWRKY25* can complement the *atwrky33* mutant when constitutively expressed (Figs 2, 3), AtWRKY33 CTD and its promoter appear to have quantitative roles in enhancing the regulatory activity of AtWRKY33 in plant defence and stress responses. Similar observations have also been made for the Arabidopsis histone H2A gene family for efficient transformation of Arabidopsis roots by *Agrobacterium tumefaciens*. Disruption of the H2A gene *HTA*1 in the Arabidopsis *rat5* mutant decreases transformation (Mysore *et al.*, 2000), which can be restored by other members of the H2A gene family if they are driven by the CaMV *35S* promoter, but not by their native promoters (Yi *et al.*, 2006). Expression analysis indicates that although these HTA genes display distinct but somewhat overlapping expression patterns in mature plants, only *HTA1* is responsive to wounding and *Agrobacterium* infection in the roots segments most susceptible to transformation (Yi *et al.*, 2002, 2006). Thus, structurally highly conserved HTA proteins have functionally diversified through distinct expression patterns controlled by regulatory promoter sequences. On the other hand, closely related WRKY proteins such as WRKY33 and WRKY25 have functionally diversified through significant diversification of both coding and non-coding regulatory sequences even though they still share a great degree of similarities in both structure and expression. The distinct but somewhat overlapping nature of both structure and expression would make it possible for complex functional interactions among closely related WRKY proteins. Indeed, a number of reported studies on closely related WRKY proteins (e.g. Arabidopsis AtWRKY18, AtWRKY40 and AtWRKY60) have revealed not only additive but also cooperative or antagonistic effects of functional interactions (Xu *et al.*, 2006; Chen *et al.*, 2010; Pandey *et al.*, 2010; Liu *et al.*, 2012), which could provide an important mechanism for elaborate regulation of a large number of genes involved in plant defence and stress responses.

### Evolutionarily conserved WRKY33 homologues in crop plants

Phylogenetic analysis of Group I proteins using whole protein sequences identified homologues of Arabidopsis AtWRKY33 in both tomato and rice (Fig. 8). These tomato and rice WRKY33 homologues are structurally closer to AtWRKY33 than any other WRKY protein from Arabidopsis, including AtWRKY25 and AtWRKY26 (Fig. 8). Sequence alignment between AtWRKY33 and the two tomato WRKY33 homologues showed extensive sequence similarity over the entire proteins including the extended CTDs (Zhou *et al.*, 2014). Silencing of *SlWRKY33A* and *SlWRKY33B* compromised tomato resistance to *Botrytis* and heat tolerance (Fig. 10) (Zhou *et al.*, 2014). Both *SlWRKY33A* and *SlWRKY33B* driven by both the CaMV *35S* promoter and the *AtWRKY33* promoter complemented Arabidopsis at*wrky33* mutant plants for *Botrytis* resistance and heat tolerance (Figs 2–5). These results indicate that WRKY33 proteins are both structurally and functionally conserved transcription factors with important roles in plant responses to both biotic and abiotic stresses.

Through phylogenetic analysis, three close homologues of Arabidopsis AtWRKY33 have also been identified in the monocot plant rice (Fig. 8). Arabidopsis AtWRKY33 is activated through phosphorylation by pathogen/stress-responsive MPK3 and MPK6 and activated AtWRKY33 is important for pathogen-induced phytoalexin biosynthesis (Mao *et al.*, 2011). A similar pathogen-responsive OsMPK3/6 cascade plays a crucial role in pathogen-induced biosynthesis of diterpenoid phytoalexins in rice (Kishi-Kaboshi *et al.*, 2010a). Although a rice bZIP transcription factor OsTGAP1 has been shown to be critical in production of diterpenoid phytoalexins in rice (Okada *et al.*, 2009), it is not phosphorylated by rice OsMPK3 or OsMPK6 (Kishi-Kaboshi *et al.*, 2010b). On the other hand, Os05g27730 (previously Os05g034300, also named OsWRKY53 or OsWRKY61), a close rice homologue of AtWRKY33 (Fig. 8), is induced by pathogen infection and pathogen elicitors, and its overexpression increases resistance to rice blast disease (Chujo *et al.*, 2007; Berri *et al.*, 2009). It is tempting to suggest that one or more rice WRKY33 proteins might also function downstream of the MAPK cascade in the regulation of phytoalexin biosynthesis. In *Nicotiana benthamiana*, another Group I WRKY protein, NbWRKY8, which shares ~80% amino acid sequence identity with tomato SlWRKY33b, is a substrate of SIPK and WIPK (orthologues of Arabidopsis MPK3 and MPK6) (Ishihama *et al.*, 2011). Phosphorylation of NbWRKY8 leads to enhanced DNA-binding and transcription-activating activities. Overexpression of a phospho-mimicking NcWRKY8 mutant induced expression of defence genes, including a gene encoding 3-hydroxy-3-methylglutuaryl CoA reductase critical for biosynthesis of isoprenoid phytoalexins (Ishihama *et al.*, 2011). Thus, NbWRKY8 is also activated through phosphorylation by stress/pathogen-responsive MAPKs and positively regulates genes involved in biosynthesis of isoprenoid phytoalexins in the solanaceous plant, supporting NbWRKY8 as a tobacco orthologue of AtWRKY33 (Ishihama *et al.*, 2011).

Pathogen-induced expression of AtWRKY33 is regulated by a composite promoter motif containing three WRKY-recognized W-box elements and activated AtWRKY33 binds to its own promoter to activate its own expression. Significantly, pathogen-responsive PcWRKY1, the parsley orthologue of AtWRKY33, has a similar composite DNA motif containing three closely spaced W boxes in its promoter and ChIP showed elicitor-dependent *in vivo* binding of parsley PcWRKY1 to the W boxes within the PcWRKY1 promoter. In addition, OsWRKY53 (Os05g27730 in Fig. 8), a close homologue of *AtWRKY33*, also contains three tandem W-box elements that are essential for the elicitor-responsiveness of the gene (Chujo *et al.*, 2009). Therefore, there is extensive conservation among the *WRKY33* gene homologues not only in the coding sequences but also in the regulatory *cis*-acting elements. Recognition of the W-box elements in these WRKY33 gene promoters by activated WRKY33 would generate a potential positive feedback mechanism for rapid and strong induction of WRKY33 genes and, consequently, WRKY33-mediated stress responses. Indeed, we have shown that *AtWRKY33* driven by the *AtWRKY25* gene promoter failed to restore resistance to *Botrytis* and could only partially restore heat tolerance of *atwrky33* mutant plants (Fig. 6). The strong conservation in the regulatory *cis*-acting elements among WRKY33 homologous genes between such evolutionarily distant plant species as Arabidopsis, parsley and rice further underscore the importance of the expression patterns conferred by the *WRKY33* gene promoters in plant stress responses.

In conclusion, WRKY33 transcription factors are highly conserved during the course of plant evolution not only in structure and function but also in the regulatory network. Therefore, plants have a highly conserved WRKY33 pathway that protects land plants against a number of important biotic and abiotic stresses. In the WRKY33 pathway, a stress signal is transmitted to WRKY33 by post-translational modifications through protein phosphorylation by a MAPK cascade and other interacting proteins such as VQ proteins. This results in the activation of WRKY33 as a transcription factor that binds to its own promoter and activates its expression. The positive autoregulatory feedback loop leads to rapid and strong induction of high levels of activated WRKY33 proteins and strong induction of WRKY33 target genes. The transcriptional network of WRKY33-responsive genes produces proteins in a wide range of biological processes associated with stress responses including production of antimicrobial compounds (e.g. phytoalexins), autophagy, JA responses and redox homeostasis. Further studies aimed at understanding the conserved WRKY33 pathway should generate important information about the general molecular mechanisms of plant stress responses.

## Supplementary data

Supplementary data are available at *JXB* online.

Supplementary Figure 1. Expression of WRKY transgenes under control of the constitutive CaMV *35S* promoter in the transgenic *atwrky33* mutant plants.

Supplementary Figure 2. TRV-mediated silencing of *SlWRKY33A* and *SlWRKY33B*.

Supplementary Figure 3. Heat-induced tomato *SlWRKY33* transgene expression conferred by the *AtWRKY33* promoter in the transgenic *atwrky33* mutant plants.

Supplementary Table 1. Primers for PCR amplification of WRKY gene promoters and coding sequences (CDS).

Supplementary Table 2. Primers for qRT-PCR.

Supplementary Data
