# Supplementary material for: Characterization of the promoter and extended C-terminal domain of Arabidopsis WRKY33 and functional analysis of tomato WRKY33 homologues in plant stress responses
Source: J Exp Bot. 2015 May 11;66(15):4567–83. doi: 10.1093/jxb/erv221 (PMC4507763; doi:10.1093/jxb/erv221)
Supplement: Supplementary Data [file supp_66_15_4567__index.html]

Characterization of the promoter and extended C-terminal domain of Arabidopsis WRKY33 and functional analysis of tomato WRKY33 homologues in plant stress responses — Characterization of the promoter and extended C-terminal domain of Arabidopsis WRKY33 and functional analysis of tomato WRKY33 homologues in plant stress responses — Supplementary Data 

# Characterization of the promoter and extended C-terminal domain of Arabidopsis WRKY33 and functional analysis of tomato WRKY33 homologues in plant stress responses

## Supplementary Data

Data files

**Files in this Data Supplement:**

- Supplementary Data - Supplementary Data
