# Supplementary material for: Characterization of the promoter and extended C-terminal domain of Arabidopsis WRKY33 and functional analysis of tomato WRKY33 homologues in plant stress responses
Source: J Exp Bot. 2015 May 11;66(15):4567–83. doi: 10.1093/jxb/erv221 (PMC4507763; doi:10.1093/jxb/erv221)
Supplement: Supplementary Data [file supp_erv221_jexbot143446_file001.pdf]

Supplemental Figure 1

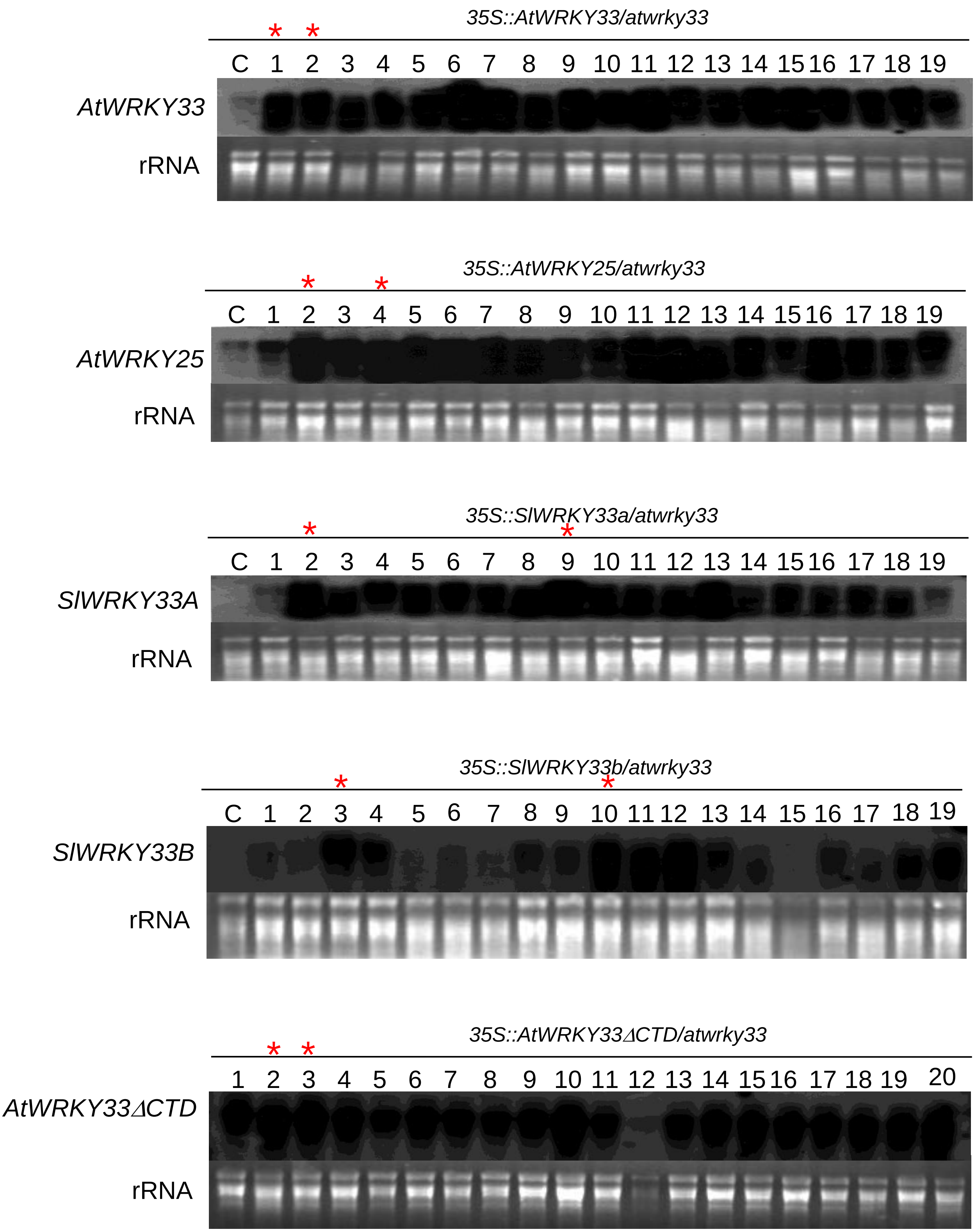

Supplemental Figure 1. Expression of WRKY transgenes under control of the constitutive CaMV *35S* promoter in the transgenic *atwrky33* mutant plants.

Total RNA was isolated from 5-week old *atwrky33* control plants (C) or independent transgenic *atwrky33* lines harboring a WRKY transgene-overexpressing construct. F2 progeny from two independent lines expressing high levels of a transgene were used for analysis of *Botrytis* resistance and heat tolerance. The transgenic lines selected for further analysis are indicated with asterisks.

Supplemental Figure 2

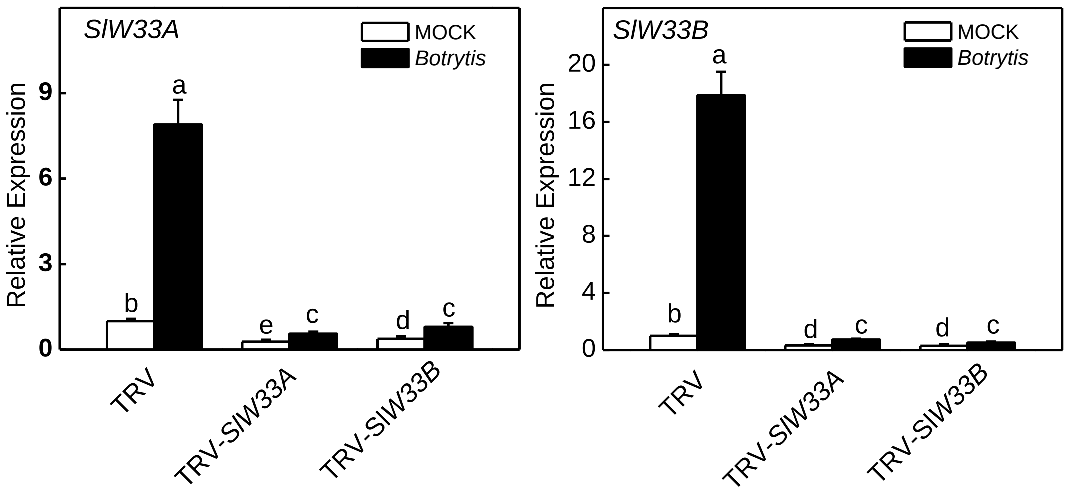

Supplemental Figure 2. TRV-mediated silencing of *SlWRKY33A* and *SlWRKY33B*. *SlWRKY33* transcript levels in mock- and *Botrytis*-inoculated tomato plants infiltrated with *Agrobacterium* cells harboring the empty TVR vector or the silencing TRV-SlWRKY33A (TRV-SlW33A) or TRV-SlWRKY33B (TRV-SlW33B) vector. Total RNA was isolated for qRT-PCR analysis from the terminal leaflets of the fourth leaves of *Agrobacterium*-infiltrated tomato plants harvested at 3 dpi. Error bars indicate SE (n = 3). According to Duncan's multiple range test (P = 0.05), means of lesion areas do not differ significantly if they are indicated with the same letter.

Supplemental Figure 3

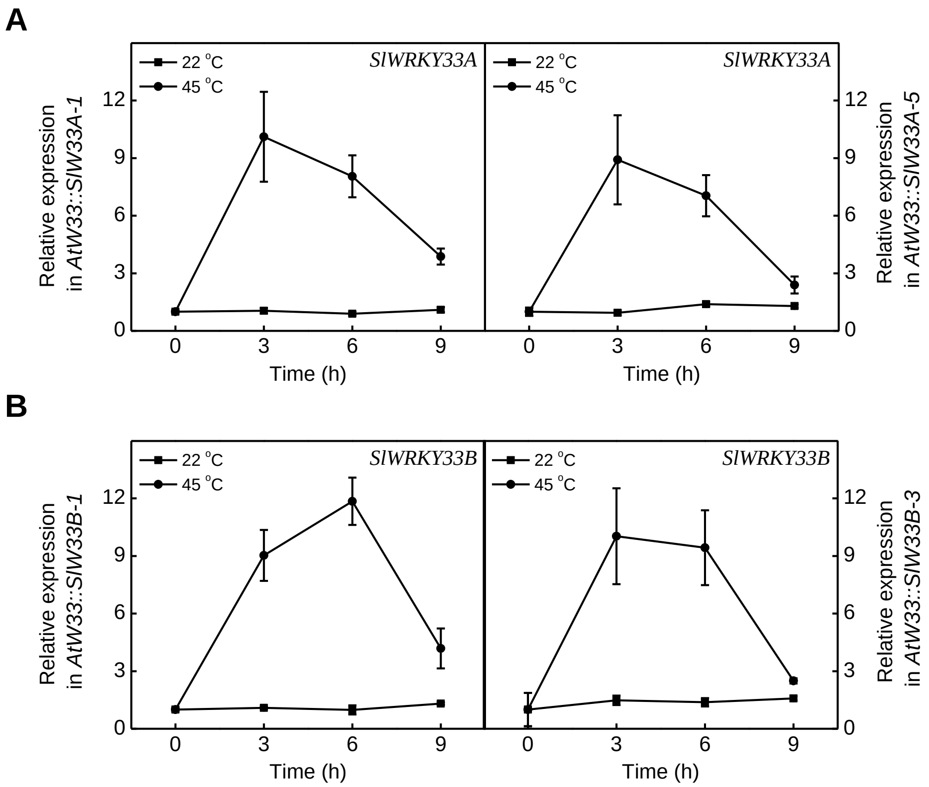

Supplemental Figure 3. Heat-induced tomato *SlWRKY33* transgene expression conferred by the *AtWRKY33* promoter in the transgenic *atwrky33* mutant plants.

Total RNA was isolated from the first and second lines of transgenic *atwrky33* mutant plants harboring the *SlWRKY33A* (A) and the first and third lines of harboring the *SlWRKY33B* (B) under the control of the *AtWRKY33* promoter (*AtW33::SlW33A* and *AtW33::SlW33B*, respectively) harvested at indicated time at 45°C. Transcript levels were determined using qRT-PCR. Error bars indicate SE (n = 3).

Supplemental Table S1. Primers for PCR amplification of WRKY gene promoters and coding sequences (CDS).

Name	Cloning Restriction sites	Primer
AtWRKY25 promoter	HidIII<br>SacI	F: 5’TTCCCAATCCATTGGTAGTCA 3’<br>R: 5’ATCGAGCTCAAAACAGAGGATTCGCTAAAAA 3’
AtWRKY33 promoter	HidIII<br>SacI	F: 5’ATCAAGCTTCCACATATCGTGCAATAAGAAACT 3’<br>R: 5’ATCGAGCTCACGAAAAATGGAAGTTTGTTTTATAAAAGA 3’
AtWRKY25 CDS	SacI<br>BamHI	F: 5’ATCGACCTCATGTCTTCCACTTCTTTCACCGA 3’<br>R: 5’ATCGGATCCTCACGAGCGACGTAGCGCGGT 3’
AtWRKY33 CDS	SacI<br>BamHI	F: 5’ATCGACCTCATGGCTGCTTCTTTTCTTACAATGG 3’<br>R: 5’ATCGGATCCAGGGCATAAACGAATCGAAAA 3’
AtWRKY33 CDS	SacI<br>BamHI	F: 5’ATCGACCTCATGGCTGCTTCTTTTCTTACAATGG 3’<br>R: 5’ATCGGATCCAAACCACGAGCTGCAGGAACATC 3’
SlWRKY33 A CDS	SacI<br>BamHI	F: 5’ATCGACCTCATGGCTGCTTCAAGTTTCTCT 3’<br>R: 5’ATCGGATCCTCGAGGCTCGTCCTTAGTTC 3’
SlWRKY33 B CDS	KpnI<br>SmaI	F: 5’ATCGGTACCATGGCTTCTTCAGGTGGAAAT 3’<br>R: 5’GCTGAAGAATAAATCATCTTTGGGTT 3’

Supplemental Table S2. Primers for qRT-PCR

GENE NAME	PRIMERS	
	FORWARD	REVERSE
AtWRKY33	TACGAAGGGAAACACAACCA	AAGGCCCGGTATTAGTGTTG
AtActin2	CTAACCTCTCTCAAGATCAAAGGCT	ACTAAAACGCAAAACGAAAGCGG
BcActin	AATGTTACCATACAAATCCTTACGGACA	ACTCATATGTTGGAGATGAAGCGCAA
SlWRKY33A	GCATTACTGTCAACCATCGC	AACTTCGCGGATTCTCACTT
SlWRKY33B	CCACAACAGTCTGAAATGGG	CAGCAAAGCAATGACTCCAT
SlActin	TGTCCCTATTTACGAGGGTTATGC	CAGTTAAATCACGACCAGCAAGAT
